# A Novel Risk-Stratification Models of the High-Flow Nasal Cannula Therapy in COVID-19 Patients With Hypoxemic Respiratory Failure

**DOI:** 10.3389/fmed.2020.607821

**Published:** 2020-12-08

**Authors:** Jiqian Xu, Xiaobo Yang, Chaolin Huang, Xiaojing Zou, Ting Zhou, Shangwen Pan, Luyu Yang, Yongran Wu, Yaqi Ouyang, Yaxin Wang, Dan Xu, Xin Zhao, Huaqing Shu, Yongxiang Jiang, Wei Xiong, Lehao Ren, Hong Liu, Yin Yuan, Hong Qi, Shouzhi Fu, Dechang Chen, Dingyu Zhang, Shiying Yuan, You Shang

**Affiliations:** ^1^Department of Critical Care Medicine, Union Hospital, Tongji Medical College, Huazhong University of Science and Technology, Wuhan, China; ^2^Research Center for Translational Medicine, Jinyintan Hospital, Wuhan, China; ^3^Department of ICU/Emergency Wuhan Third Hospital, Wuhan University, Wuhan, China; ^4^Department of Pulmonary and Critical Care Medicine, Ruijin Hospital, Shanghai Jiao Tong University School of Medicine, Shanghai, China; ^5^Institute of Anesthesiology and Critical Care Medicine, Union Hospital, Tongji Medical College, Huazhong University of Science and Technology, Wuhan, China

**Keywords:** COVID-19, HFNC, ROX, mechanical ventilation, thrombocytopenia, risk-stratification

## Abstract

**Background:** High-flow nasal cannula (HFNC) has been recommended as a suitable choice for the management of coronavirus disease 2019 (COVID-19) patients with acute hypoxemic respiratory failure before mechanical ventilation (MV); however, delaying MV with HFNC therapy is still a dilemma between the technique and clinical management during the ongoing pandemic.

**Methods:** Retrospective analysis of COVID-19 patients treated with HFNC therapy from four hospitals of Wuhan, China. Demographic information and clinical variables before, at, and shortly after HFNC initiation were collected and analyzed. A risk-stratification model of HFNC failure (the need for MV) was developed with the 324 patients of Jin Yin-tan Hospital and validated its accuracy with 69 patients of other hospitals.

**Results:** Among the training cohort, the median duration of HFNC therapy was 6 (range, 3–11), and 147 experienced HFNC failure within 7 days of HFNC initiation. Early predictors of HFNC failure on the basis of a multivariate regression analysis included age older than 60 years [odds ratio (OR), 1.93; 95% confidence interval (CI), 1.08–3.44; *p* = 0.027; 2 points], respiratory rate-oxygenation index (ROX) <5.31 (OR, 5.22; 95% CI, 2.96–9.20; *p* < 0.001; 5 points) within the first 4 h of HFNC initiation, platelets < 125 × 10^9^/L (OR, 3.04; 95% CI, 1.46–6.35; *p* = 0.003; 3 points), and interleukin 6 (IL-6) >7.0 pg/mL (OR, 3.34; 95% CI, 1.79–6.23; *p* < 0.001; 3 points) at HFNC initiation. A weighted risk-stratification model of these predictors showed sensitivity of 80.3%, specificity of 71.2% and a better predictive ability than ROX index alone [area under the curve (AUC) = 0.807 vs. 0.779, *p* < 0.001]. Six points were used as a cutoff value for the risk of HFNC failure stratification. The HFNC success probability of patients in low-risk group (84.2%) was 9.84 times that in the high-risk group (34.8%). In the subsequent validation cohort, the AUC of the model was 0.815 (0.71–0.92).

**Conclusions:** Aged patients with lower ROX index, thrombocytopenia, and elevated IL-6 values are at increased risk of HFNC failure. The risk-stratification models accurately predicted the HFNC failure and early stratified COVID-19 patients with HFNC therapy into relevant risk categories.

## Introduction

Severe coronavirus disease 2019 (COVID-19) caused by severe acute respiratory syndrome coronavirus 2 (SARS-CoV-2) infection was characterized by progressive dyspnea and hypoxemia within 1 week after onset of the disease (1–4). As of August 23, 2020, more than 1.7 million new COVID-19 patients were reported in the last 7 days, by the World Health Organization Region. For most patients, they recovered on conventional oxygen therapy. However, some patients, mainly critically ill patients, progressed to severe respiratory distress, and needed advanced oxygen therapy, including high-flow nasal cannula (HFNC) therapy and mechanical ventilation (MV) (2).

HFNC can bring high concentrations of humidified oxygen with low level of positive end-expiratory pressure; facilitate the elimination of carbon dioxide, allowing one to rapidly relieve the acute hypoxemic respiratory failure (AHRF) symptoms (5, 6); and apply in critical care resource-limited countries. During the ongoing pandemic, HFNC has been recommended as a bridge for management of patients with severe or critical COVID-19 on the basis of the evidences from non-coronavirus-related pneumonia (6–9). To our best knowledge, however, one important concern during HFNC therapy in non-COVID-19 patients with AHRF is to not delay the need of MV (10), and the association between HFNC therapy and its outcome (need or not for MV) and its early predictors has not been explored.

Here, we reported the clinical course of COVID-19 patients receiving HFNC and explored the predictors and attempted to develop a novel risk-stratification model that predicts the failure (the need for MV) of HFNC therapy for COVID-19 patients and early stratifies them into relevant risk categories.

## Methods

### Study Design and Participants

This was a multicenter retrospective observational study on adult COVID-19 patients receiving HFNC oxygen therapy in Union Hospital, Wuhan Third Hospital, Union Hospital West Campus, or Jinyintan Hospital between December 29, 2019, and April 30, 2020. The training cohort was set up in Jin Yin-tan Hospital, and the validation cohort was set up in the other hospitals. All patients with a diagnosis of COVID-19 according to guidelines released by the National Health Commission of the People's Republic of China were screened (11). Patients were excluded if they never received HFNC, received HFNC after MV, deceased within 12 h after admission, had important information that was missing within 12 h of HFNC therapy, or were included in previous studies (2, 4, 12, 13). Research approval (2020-0041-1) was granted by the ethics board of Wuhan Union Hospital as the central coordinating center. The need for informed consent was waived.

### HFNC Device and Treatment

HFNC was considered if a patient with COVID-19 required a conventional oxygen therapy with oxygen flow higher than 10 L/min to achieve Spo_2_ of >90% or a respiratory rate (RR) >30 breaths per min (bpm) despite adequate oxygen supplementation or showed persistent signs of respiratory distress (10, 14–17).

The included COVID-19 patients received HFNC oxygen therapy via HFNC device (Fisher & Paykel, Auckland, New Zealand). The temperature of the heated humidification system was adjusted between 31 and 37°C to provide optimal humidity; the flow was initiated with a minimum flow at 30 L/min, and Fio_2_ was adjusted to reach Spo_2_ >90% or higher (10, 14, 18, 19).

The discontinuation of HFNC oxygen therapy and the switch to either conventional oxygen therapy or MV, was made at the discretion of the treating physicians. If respiratory failure improved, a trial of intermittent HFNC therapy was performed, and the duration of HFNC was gradually shortened until the HFNC was totally substituted by conventional oxygen therapy. If the respiratory failure progressively deteriorated, non-invasive or invasive MV was used. The decision to intubate was at the discretion of the treating physicians in accordance with published guidelines [26].

### Data Collection

Patient identification in these ICUs was achieved by reviewing admission logs from available medical records. After several cycles of feedback and pilot testing, modified standardized International Severe Acute Respiratory and Emerging Infection Consortium case report forms for COVID-19 were utilized (12). Data were extracted from local servers by experienced research physicians at each center.

Demographic data, preexisting comorbidities (chronic cardiac disease, chronic pulmonary disease, hypertension, cerebrovascular disease, chronic liver disease, diabetes, and malignancy), vital signs (RR, heart rate, blood pressure), and laboratory values [white blood cell count, platelets, prothrombin time (PT), activated partial thromboplastin time (APTT), plasma d-dimer, plasma fibrinogen, alanine aminotransferase (ALT), aspartate aminotransferase (AST), serum creatinine, hypersensitive troponin I (hsTNI), IL-6, arterial blood gas analysis], complications [acute cardiac injury, acute kidney injury (AKI), liver dysfunction, coagulopathy, and hospital acquired infection], and treatments (oxygen therapy, corticosteroids) were collected at admission and HFNC and MV initiation.

### Definitions

AKI was diagnosed based on the serum creatinine criterion of KDIGO (20). Acute cardiac injury was diagnosed if the serum concentration of hsTNI was measured in the laboratory above the upper limit of the reference range (>28 pg/mL) (12). Liver dysfunction was diagnosed if serum ALT >50 U/L or AST >40 U/L (21). Coagulopathy was defined if PT >16.5 s or APTT >42 s (13). Shock was defined according to the 2016 Third International Consensus Definition for Sepsis and Septic Shock (22). Respiratory rate–oxygenation (ROX) was defined as the ratio of Spo_2_/Fio_2_ to respiratory rate (18, 23). HFNC therapy failure was defined as need for MV within 7 days of HFNC initiation (24). The criteria for typical HFNC failures include failures to maintain Spo_2_ >90%, hypercapnic respiratory failures with pH <7.2 or Paco_2_ of arterial blood >55 mm Hg, worsening respiratory distress, or need for airway protection due to altered mental state or aspiration (10, 14, 15, 18, 19).

### Statistical Analysis

No hypothesis was made for the present study, so sample size estimation was unavailable. Data were expressed as mean ± standard deviation, median [interquartile range (IQR)] or median (range) for continuous variables, and number (%) for categorical variables. Differences between patients with failure and success of HFNC oxygen therapy, and between survivors and non-survivors of MV after HFNC failure, were explored using two-sample *t*-test for parametric variables, Wilcoxon rank-sum test for non-parametric variables, and Fisher exact test for categorical variables. Age was dichotomized at 60 years (25). IL-6, d-dimer, and platelet count were dichotomized at the clinically relevant cutoff. ROX index was also included and dichotomized at the optimal cutoff point following Youden index of receiver operating characteristic (ROC) curve (18). Dichotomized age, IL-6, platelet counts, comorbidities, and complications showing a *p* < 0.1 in univariate analysis were included for multiple logistic regression analysis. The scores were assigned as integer values relative to the regression coefficient of each predictor. The predictive value of the risk-stratification models was assessed by the area under the curve (AUC). Cutoff points were calculated according to Youden index of ROC. Success curves of HFNC oxygen therapy between low-risk and high-risk of HFNC failure following the cutoff value and survival analysis for the patients who received MV before and after 48 h of HFNC initiation were developed using the Kaplan–Meier method.

All statistical tests were 2-tailed with significance set at *p* < 0.05. The Stata/IC 15.1 software (StataCorp, College Station, TX, USA) was applied for all analyses.

## Results

### Demographic Data and Clinical Parameters of Included Patients

We screened 3,102 patients with confirmed COVID-19 between December 29, 2019, and March 30, 2020, of which 546 patients (17.6%) with severe AHRF were identified, and 324 patients were included into training cohort, and 69 patients formed the validation cohort.

Among the training cohort, the mean age of COVID-19 patients was 63.2 ± 14.5 years, of whom 211 patients (65.1%) were older than 60 years, and 219 (67.6%) were male (Table 1 and Supplementary Table 1). The proportion of patients with coexisting conditions were not significantly different between the HFNC success group and the HFNC failure group except for malignancy (*p* = 0.024). The ratio of positive SARS-CoV-2 RNA at HFNC initiation and the duration of HFNC initiation to the negative conversion of SARS-CoV-2 RNA for the survivors was not different between the two groups.

**Table 1:** Baseline characteristics of the included 324 patients with COVID-19 at HFNC initiation.

**Characteristics**	**All patients (*n* = 324)**	**HFNC success (*n* = 177)**	**HFNC failure (*n* = 147)**	***p*-value success vs. failure**
Age, mean ± SD, years	63.2 ± 14.5	60.6 ±15.5	66.3 ± 12.5	0.0005
Age ≥60 years	211 (65.1%)	99 (55.9%)	112 (76.2%)	<0.001
Male	219 (67.6%)	119 (67.2%)	100 (68.0%)	0.487
**Preexisting comorbidities**				
Hypertension	147 (45.4%)	78 (44.1%)	69 (46.9%)	0.343
Diabetes	60 (18.5%)	34 (19.2%)	26 (17.7%)	0.419
Chronic cardiac disease	42 (13.0%)	22 (12.4%)	20 (13.6%)	0.440
Chronic pulmonary disease	26 (8.0%)	13 (7.3%)	13 (8.8%)	0.385
Chronic liver disease	27 (8.4%)	14 (8.0%)	13 (8.8%)	0.464
Cerebrovascular disease	25 (7.7%)	10 (5.7%)	15 (10.2%)	0.094
Malignancy	15 (4.6%)	4 (2.3%)	11 (7.5%)	0.024
Time from illness onset to HFNC, days	14 [10–19]	14 [11–19]	14 [10–18]	0.7615
Time from admission to HFNC, days	2 [0–5]	2 [1–4]	2 [0–6]	0.5397
SOFA at HFNC onset (n = 218)[Table-fn TN1]	3 [2–4]	2 [2–3.5]	4 [3–5]	<0.001
**Laboratory findings at HFNC initiation**				
SARS-CoV-2 RNA positive	108 (33.3%)	64 (36.2%)	44 (29.9%)	0.143
Time to negative of SARS-CoV-2 RNA, days[Table-fn TN2]	6 [0–16]	7.5 [0–16]	5.5 [0 −6]	0.1612
Neutrophils, 10^9^/L	7.9 [5.6–11.1]	7.4 [5.3–9.8]	8.5 [6.0–11.8]	0.0058
Neutrophils >6.3 × 10^9^/L^||^	220 (67.9)	114(64.4%)	106 (72.1%)	0.087
Lymphocytes, 10^9^/L	0.6 [0.4–0.9]	0.6 [0.4–0.9]	0.6 [0.4–0.8]	0.2715
Platelets 10^9^/L	187 [140.5–246.5]	200 [149–255]	177 [119–234]	0.0087
Platelets <125 × 10^9^/L^‡^	65 (20.1%)	22(12.4%)	43 (29.3%)	<0.001
D-Dimer, μg/mL	3.3 [1.0–14.1]	2.6 [0.8–8.8]	4.8[1.1–17.7]	0.0203
Elevated d-dimer level^**†**^	210 (64.8%)	109 (61.6%)	101 (68.7%)	0.111
Fibrinogen, g/L	4.8 [3.4–6.1]	4.8 [3.2–6.6]	4.8 [3.6–5.8]	0.5825
IL-6^¶^, pg/mL	9.7 [7.0–14.5]	9.6 [6.6–14.5]	10.0 [7.8–14.7]	0.1509
Elevated IL-6 level l^¶^	223 (68.8%)	99 (55.9%)	124 (84.4%)	<0.001
**Complications at HFNC initiation**				
Shock	2 (0.62%)	0 (0.0%)	2(1.36%)	0.36
AKI	37 (11.4%)	11 (6.2%)	26 (17.7%)	0.001
Acute cardiac injury	188 (58.0%)	91 (51.4%)	97 (66.0%)	0.006
Liver dysfunction	189 (58.3%)	97 (54.8%)	92 (62.6%)	0.096
Coagulopathy	30 (9.3%)	12(6.8%)	18 (12.2%)	0.067

*AKI, acute kidney injury; COVID-19, coronavirus disease 2019; IQR, interquartile range; SD, standard deviation; HFNC, high-flow nasal cannula; IL-6, interleukin 6; SOFA, Sequential Organ Failure Assessment*.

*Data were expressed median [interquartile range] or as mean ± standard deviation*.

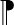
*SOFA scores at HFNC initiation were available in 218 patients, because arterial blood gas analysis was conducted in 132 HFNC failure patients and 86 success patients*.

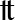
*Durations of negative conversion of SARS-CoV-2 RNA were available in 136 survivors, of 126 survivors from HFNC success group and 10 from HFNC failure group*.

|| *The upper limit of normal range of neutrophil count was 6.3 × 10^9^/L*.

† *The upper limit of normal range of d-dimer was 1.5 μg/mL*.

‡ *The lower limit of normal range of platelet count was 125 × 10^9^/L*.

¶ *The upper limit of normal range was 7 pg/mL*.

At HFNC initiation, compared with patients in the HFNC success group, the HFNC failure group had a higher level of d-dimer [4.8 (1.1–17.7) vs. 2.6 (0.8–8.8); *p* = 0.0203] and more patients with IL-6 >7.0 pg/mL [124 (84.4%) vs. 99 (55.9%); *p* < 0.001] and with platelets <125 × 10^9^/L [22 (12.4%) vs. 43 (29.3%), *p* < 0.001], but no differences in eosinophils, lymphocytes, the proportion of elevated d-dimer, and concentration of plasma fibrinogen and IL-6 were observed between the two groups. Moreover, there was a higher prevalence of AKI [26 (17.7%) vs. 11 (6.2%); *p* = 0.001] and acute cardiac injury [97 (66.0%) vs. 91 (51.4%); *p* = 0.006] in the HFNC failure group, but no differences in shock, liver dysfunction, coagulopathy, and corticosteroids treatment were observed between the two groups.

### Respiratory Variables During HFNC Treatment of the Training Cohort

At baseline, 218 patients had analyses of arterial blood gas, and the number of patients with Pao_2_/Fio_2_ <200 mm Hg was 174. The patients in the HFNC failure group had lower levels of Pao_2_/Fio_2_ [129.6 (100.0–163.4) vs. 151.2 (122.8–199.2) mm Hg; *p* < 0.001].

After HFNC therapy, the patients in the HFNC success group had higher Spo_2_/Fio_2_ [134.2 (117.5–158.3) vs. 108.7 (94.6–116.3) fold; *p* < 0.001] and lower RR [22 (20–24) vs. 23 (22–25); *p* < 0.001] (Table 2), but no differences in systolic arterial pressure were observed between the two groups. The RR alone had an AUC of 0.65 (0.59–0.71). Higher ROX index values with an AUC of 0.779 (0.73–0.83) were observed in the HFNC success group [5.9 (5.0–7.5) vs. 4.8 (4.1– 5.3); *p* < 0.001]. The optimal cutoff point for the ROX index within the first 4 h was estimated to be 5.31 in accordance to the ROC curve, more patients in the HFNC failure group had an ROX index score <5.31 [114 (77.6%) vs. 60 (33.9%), *p* < 0.001].

**Table 2:** Physiologic variables at HFNC initiation and 12 h of HFNC onset.

**Parameters**	**All patients (*n* = 324)**	**HFNC success (*n* = 177)**	**HFNC failure (*n* = 147)**	***p*-value success vs. failure**
Heart rate at HFNC initiation	88.8 ± 16.3	87.0 ± 13.8	91.0 ± 18.7	0.0121
Systolic arterial pressure at HFNC initiation	129.2 ± 17.4	128.3 ± 17.0	130.3 ± 17.8	0.1612
**Arterial blood gas analysis at HFNC initiation** [Table-fn TN7]				
pH	7.47 ± 0.05	7.46 ± 0.04	7.47 ± 0.07	0.1103
Paco_2_, mm Hg	34.3 ± 7.7	34.9 ± 6.5	33.5 ± 9.1	0.0106
${\text{HCO}}_{3}^{-}$, mmol/L	25.1 ± 5.4	25.6 ± 4.8	24.3 ± 6.1	0.1430
Pao_2_/Fio_2_, mm Hg	141.1 [115.6–188.9]	151.2 [122.8–199.2]	129.6 [100.0–163.4]	<0.001
<200 mm Hg	174 (79.8%)	96 (75.0%)	78 (86.7%)	0.025
**Respiratory parameters at HFNC initiation**				
Spo_2_	86 [80–90]	88 [83–92]	84 [75–89]	<0.001
Spo_2_/Fio_2_	108.7 [91.6–143.3]	121.4 [100.0–150.0]	97.8 [85.4–112.5]	<0.001
RR (beats per minute)	25 [22–30]	24 [22–30]	26 [22–30]	0.1319
ROX index	4.3 [3.4–5.5]	4.9 [3.8–6.1]	3.8 [3.0–4.7]	0.0001
**After HFNC treatment**				
Spo_2_	93 [90–96]	95 [92-96]	92 [89–94]	<0.001
Spo_2_/Fio_2_	117.5 [105.0–141.0]	134.2 [117.5–158.3]	108.7 [94.6–116.3]	<0.001
RR (beats per minute)	22 [21–24]	22 [20–24]	23 [22–25]	<0.001
Flow (L/min)	55.0 [50.0–60.0]	50.0 [50.0–60.0]	60.0 [50.0–60.0]	0.0005
ROX index	5.2 [4.6–6.4]	5.9 [5.0–7.5]	4.8 [4.1– 5.3]	<0.001
Length of HFNC, median [IQR], days	6.0 [3.0–11.0]	10.0 [7.0–15.0]	3.0 [1.0–4.0]	<0.001
Median time to MV, days^¶^	3 [1–6]	11 [9–18]	2 [1–4]	<0.001

*COVID-19, coronavirus disease 2019; Fio_2_, fraction of inspired oxygen; IQR, interquartile range; Pao_2_, partial pressure of oxygen; Paco_2_, partial pressure of carbon dioxide. ROX, Respiratory rate-oxygenation; SD, standard deviation; Data were expressed median [interquartile range] or as mean ± standard deviation*.

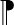
*Arterial blood gas analysis was conducted in 90 HFNC failure patients and 128 success patients*.

¶ *Median time to MV were available in 141 patients, because do-not-intubate or non-invasive ventilation intolerance was 32 in HFNC failure patients and 23 in success patients*.

### The Outcomes of HFNC Therapy of the Training Cohort and Its Predictors

The median duration of HFNC therapy was 6 (range, 3–11). One hundred forty-seven (46.4%) experienced HFNC failure within 7 days of HFNC initiation.

**Figure 1:**
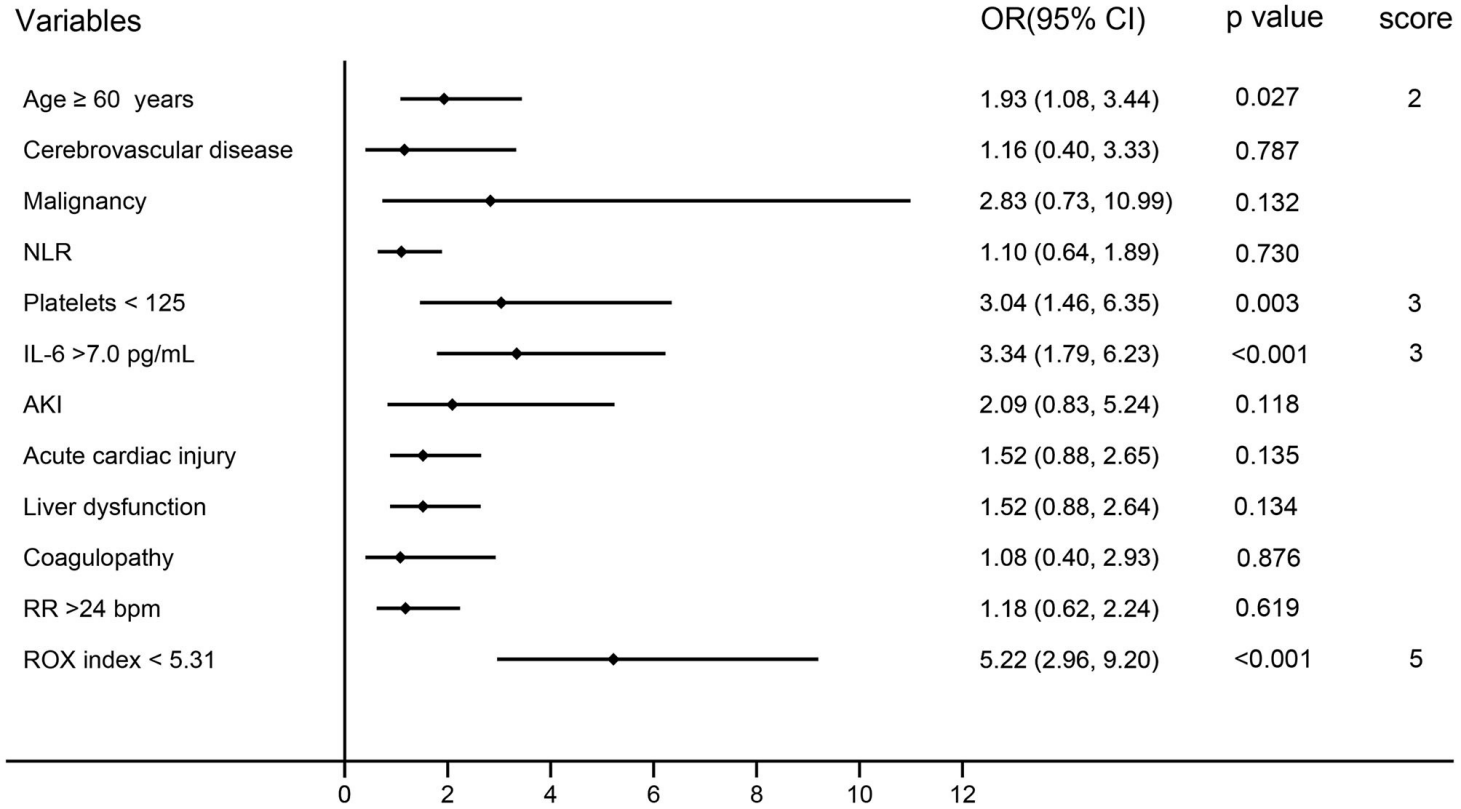
Multivariate analysis and risk-stratification models of HFNC failure in COVID-19 patients. AKI, acute kidney injury; CI, confidence interval; HFNC, high-flow nasal cannula; IL-6, interleukin 6; NLR, neutrophil-to-lymphocyte ratio; OR, odds ratio; PLT, platelets; ROX, respiratory rate-oxygenation; RR, respiratory rate.

Multiple logistic regression analysis revealed the independent predictors of HFNC failure (Figure 1), comprising age older than 60 years [odds ratio (OR) 2.54; 95% CI, 1.39–4.65; *p* = 0.003], platelets <125 × 10^9^/L (OR, 3.18; 95% CI, 1.36–7.46; *p* = 0.008), and IL-6 >7.0 pg/mL (OR, 3.07; 95% CI, 1.67–5.62; *p* < 0.001;) at HFNC initiation, ROX index <5.5 (OR, 5.92; 95% CI, 3.31–10.58, *p* < 0.001) within the first 4 h of HFNC therapy.

### Risk-Stratification Models of HFNC Therapy in COVID-19 Patients

A special risk-stratification model with relative weights according to the regression coefficient of each independent predictors is shown in Figure 1. Patients with older than 60 years were assigned a score of 2; platelets >125 × 10^9^/L and IL-6 >0.7 pg/mL were given a score of 3; ROX index <5.31 was assigned a score of 5.

**Figure 2:**
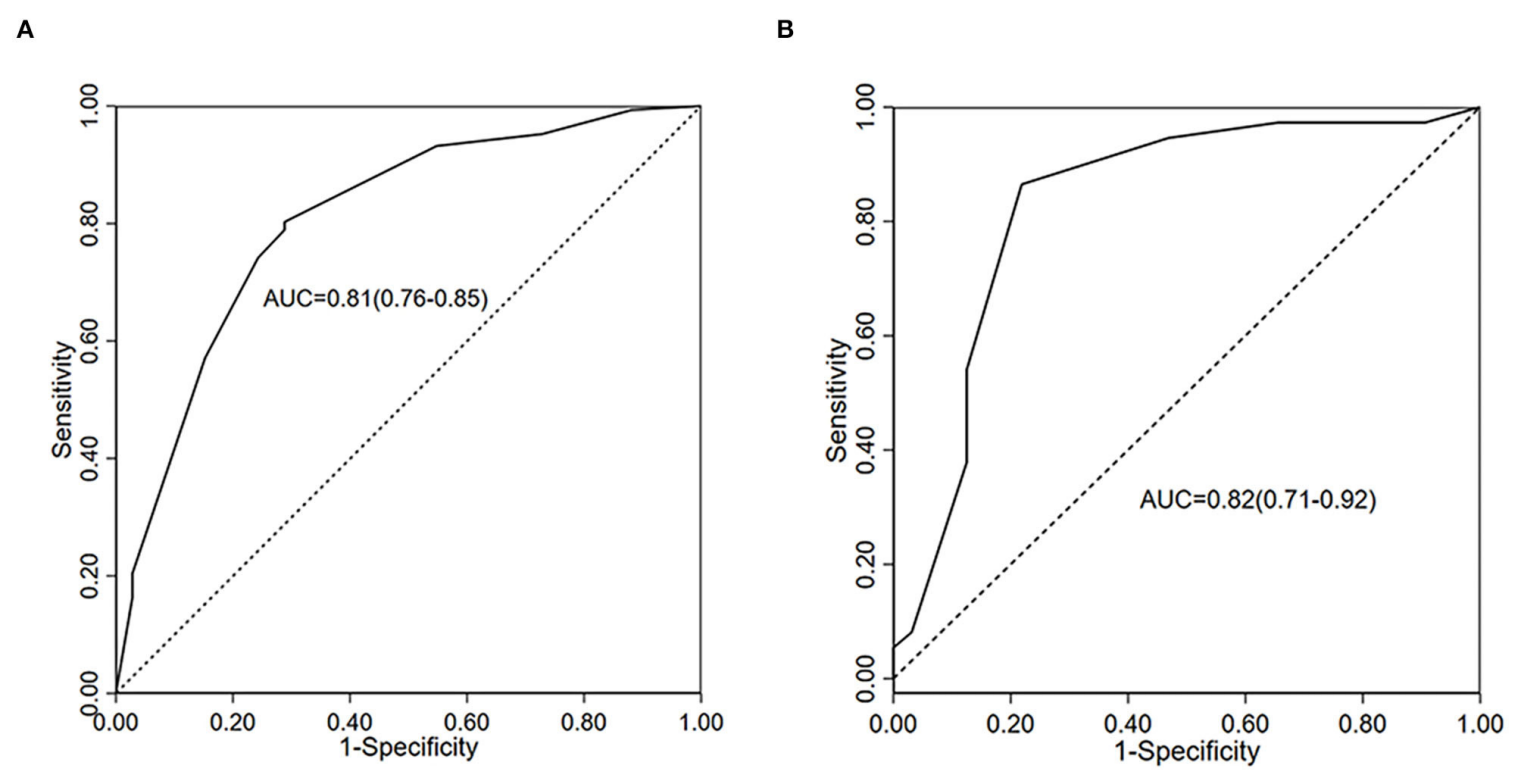
ROC curves for prediction of HFNC failure in COVID-19 patients. **(A)** AUC of the full regression model in train cohort (*n* = 324); **(B)** AUC of the Validation cohort (*n* = 69). COVID-19, coronavirus disease 2019; HFNC, high-flow nasal cannula; ROC, receiver operator characteristic; AUC, area under the curve.

The models had an AUC of 0.807 (0.76–0.85), 80.3% sensitivity, 71.2% specificity, and a greater accuracy than the ROX index alone (AUC = 0.779) in predicting the HFNC failure for COVID-19 patients (Figure 2A). The cutoff value of the models for the risk of HFNC failure stratification was six points.

**Figure 3:**
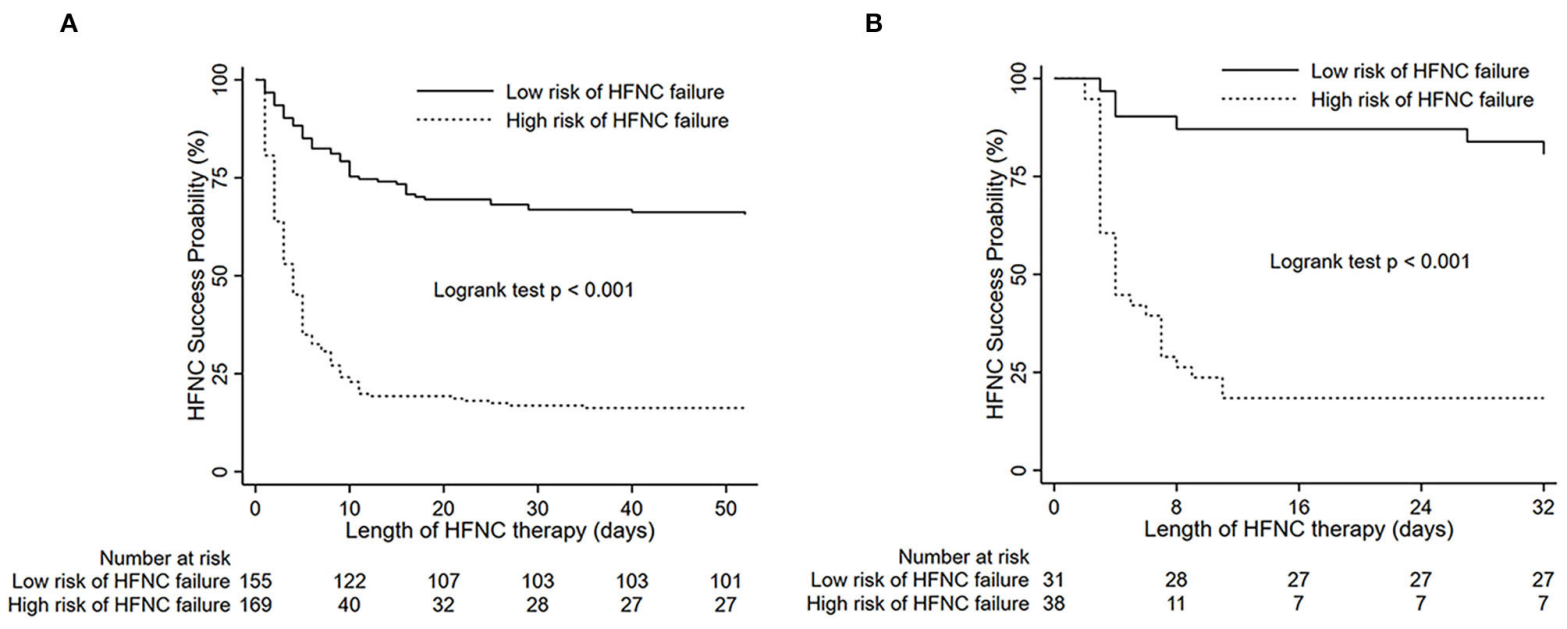
Success curves of HFNC therapy in patients from HFNC onset to termination according to different levels of score. **(A)** Training cohort; **(B)** validation cohort; For HFNC failure in hospital: weighted score 0–6 = low risk; ≥6 = high risk. HFNC, high-flow nasal cannula.

For all patients allocated into training cohort, 55 patients were deceased on HFNC therapy due to do-not-intubate (excluded from the further analysis); 142 (60.5%) patients required MV. The HFNC success probability (84.2%) for the patients who were divided into low-risk groups following the models was 9.84 times that in the high-risk group (34.8%) in hospital (Figure 3A).

### External Validation of the Models in COVID-19 Patients With HFNC Therapy

Sixty-nine patients with COVID-19 were included into validation cohort. Baseline characteristics of the patients are shown in Supplementary Tables 1, 2, and there was no significant difference in most characteristics between the two cohorts.

Twenty-six (34.7%) patients experienced HFNC failure within 7 days of HFNC initiation. The AUC of the novel models is 0.815 (0.70–0.93) (Figure 2B), sensitivity of prediction is 83.8%, and the specificity is 78.1% in prediction of HFNC failure on the basis of the variables from the scoring system. The HFNC success probability of patients is shown in Figure 3B.

## Discussion

Early identification of COVID-19 patients with AHRF during HFNC therapy at risk of failure will be beneficial for optimal use of medical resources. This retrospective cohort study on COVID-19 patients with AHRF showed that age older than 60 years, thrombocytopenia, elevated levels of IL-6 at HFNC initiation, and an ROX index <5.31 within the first 4 h of HFNC therapy initiation were independent predictors of HFNC failure. The risk-stratification model developed for HFNC therapy early predicts the need for MV with greater accuracy than the ROX index alone in COVID-19.

To our knowledge, this report was the largest cohort study to date regarding COVID-19 patients with HFNC therapy. Of patients with analyses on arterial blood gas, the median Pao_2_/Fio_2_ of 141.1 (IQR, 115.6–188.9) indicated the severity of respiratory failure. The HFNC functioned as a bridge between conventional oxygen therapy and MV (8, 16, 19). In our cohort, 36.7% patients with severe AHRF were successfully weaned from HFNC, which provided evidence to the recommendations of a trial of HFNC for COVID-19 patients with moderately severe hypoxemia to avoid the need for MV from recent WHO and other guidelines (7, 8, 26). It is reasonable that COVID-19 patients with less severe hypoxemia are more likely to wean from HFNC. In a study of 17 COVID-19 patients with HFNC in Chongqing, China, compared with 10 HFNC success patients with Pao_2_/Fio_2_ of 209 (IQR, 179–376) mm Hg, the seven HFNC failure patients had a median Pao_2_/Fio_2_ of 159 (IQR, 137–188) mm Hg (19). In another study, all the eight COVID-19 patients with a baseline mean Pao_2_/Fio_2_ of 259.88 mm Hg weaned from HFNC successfully and discharged from the hospital subsequently (27).

The global COVID-19 pandemic has led to an exploding demand for ventilators and medical service worldwide, for which many nations, including China, United States, United Kingdom, and Australia, struggled (28, 29). In face of severe shortage of ventilators and health care workers, a trial of HFNC treatment seems plausible, but brings new problems. Some patients may suffer from sudden respiratory arrest and even death during the trial, especially older patients. Our finding of variables derived before, at, and shortly after HFNC initiation predicting its success or failure is very promising to balance the need and the risk associated with the trial.

Tests on peripheral neutrophil count, thrombocyte count, and plasma IL-6 level are commonly conducted. Higher numbers of neutrophils in the peripheral blood correlated with the severity of lung damage (30). In our cohort, patients in the HFNC failure group had significantly higher neutrophil counts than those in the HFNC success group, but no difference in the proportion of neutrophilia was observed between the two groups. Most likely, of these COVID-19 patients with HFNC therapy, the severity of lung damage was comparable. In unselected COVID-19 patients, a low lymphocyte count indicates poor outcome, typically higher mortality. However, the lymphocyte counts were not different between patients in HFNC failure group and in the HFNC success group in our study. The primary reason is that the patients in our study were specifically selected. Only critically ill patients were included, and their median lymphocyte count was 0.6 × 10^9^/L, which was the same as that in the non-survivors previously reported (12, 13, 31). To explore the difference in lymphocyte count between the critically ill and the extremely critically ill, a cohort of much larger sample size will be needed. Elevated levels of IL-6, which appears to be from myeloid cells in COVID-19 patients, could predict the severity of the disease and the need for intensive care (32, 33). It occurred in 68.8% of patients in the present study, and the HFNC failure group had more patients with IL-6 above the upper limit of normal range.

ROX index, defined as the ratio of Spo_2_/Fio_2_ to RR, is easily measured at bedside, which has been considered a better predictor of HFNC success compared with Spo_2_/Fio_2_ or RR alone when measured at 2, 6, or 12 h after HFNC initiation in patients with severe community or hospital-acquired pneumonia (18, 23). ROX index ≥4.88 measured within 2–12 h of HFNC therapy was associated with increased likelihood of HFNC success in non-virus pneumonia (18, 23). In our study, COVID-19 patients who had an ROX index ≥5.31 within the first 4 h of HFNC therapy were less likely to need MV.

Advanced age, comorbidities, neutrophilia, and organ and coagulation dysfunction were statistically associated with the development of acute respiratory distress syndrome (ARDS) for COVID-19 patients, which is a determinant of the outcome of HFNC therapy (4); it is thus not reasonable to deny the important of characteristics and clinical parameters. Our novel scoring system covering COVID-19 patients' conditions, physiological variables, and laboratory detection had better predictive capacity in comparison with ROX index alone. Moreover, our scoring system shows promise for the early risk stratification of COVID-19 patients with HFNC therapy after appropriate weight.

MV is the rescue therapy for severe AHRF after HFNC failure (5, 7, 10, 34). In our study, among the patients receiving subsequent MV, 78.5% of these patients were deceased. When comparing the COVID-19 patients who received MV before and after 48 h of HFNC, there are no significant differences in 90-day mortality (Supplementary Figure 1). The proportion of patients who received the MV was different from the HFNC failure rate of patients within 7 days of HFNC onset. We postulated that the primary contributor for the difference was the progressive pathophysiological processes.

During the relatively early stage in patients with severe COVID-19, platelets take part in the formation of pulmonary microthrombi to block the viral invasion into bloodstream when SARS-CoV-2 infects the lung (35, 36). The pathophysiological process causes thrombocytopenia and elevation of IL-6, a key proinflammatory cytokine in thromboinflammatory processes (37–39). In general, pulmonary microthrombi lead to shunt in the lung vasculature, but not to decrease in lung volume, which explains the development of atypical ARDS, and so-called silent hypoxemia (40–42).

This study had several limitations. First, during the pandemic of COVD-19, oxygen therapy was the cornerstone of treating severe and critically ill patients, on which it was hard to conduct randomized controlled study. Our study was a retrospective study from a severely stricken place by the disease. The findings were at least informative to physicians from similar locations. Second, some important data, for example, Pao_2_/Fio_2_, were not available for all the patients and were not included in the regression model. However, all the identified predictors were readily accessible, except for places with extremely limited resources. Third, the sample size is not large enough, and we are expecting further studies.

## Conclusions

Thrombocytopenia, elevated levels of IL-6 at HFNC initiation, and an ROX index <5.31 within the first 4 h of HFNC therapy were independent predictors of HFNC failure, and the risk-stratification model on the basis of the four parameters, has a strong predictive ability for the need for MV in COVID-19 patients with HFNC therapy and can further classify the risk of HFNC failure. The mortality of HFNC failure patients who received MV before and after 48 h of HFNC therapy was not associated with a worse prognosis. A practical oxygen therapy for severe COVID-19 based on our findings may be proposed to prevent from or relieve the overwhelmed health care systems in resource-limited countries, where ICU devices and techniques may not be available or ICU care cannot be provided.

## Data Availability Statement

The original contributions presented in the study are included in the article/Supplementary Material. Further inquiries can be directed to the corresponding author/s.

## Ethics Statement

The studies involving human participants were reviewed and approved by Wuhan Union Hospital (2020-0041-1). Written informed consent for participation was not required for this study in accordance with the national legislation and the institutional requirements.

## Author Contributions

JX, XY, CH, XZo, TZ, SP, LY, YWu, YO, YWa, HQ, and SF collected the epidemiological and clinical data. JX, XY, XZh, WX, DX, LY, and LR summarized all data. JX, XY, LY, YY, HL, YS, and SF contributed to literature search and drafted the manuscript. DC, SY, DZ, and YS designed the study, have full access to all the data and take full responsibility for accuracy of the analyses and their interpretation. All authors contributed to the article and approved the submitted version.

## Conflict of Interest

The authors declare that the research was conducted in the absence of any commercial or financial relationships that could be construed as a potential conflict of interest.
