# Supplementary material for: A Novel Risk-Stratification Models of the High-Flow Nasal Cannula Therapy in COVID-19 Patients With Hypoxemic Respiratory Failure
Source: Front Med (Lausanne). 2020 Dec 8;7:607821. doi: 10.3389/fmed.2020.607821 (PMC7793962; doi:10.3389/fmed.2020.607821)
Supplement: Supplementary file 1 [file Data_Sheet_1.PDF]

Supplementary information

A Novel Risk-Stratification Models of the High-flow Nasal Cannula Therapy in COVID-19 patients with Hypoxemic Respiratory Failure

Jiqian Xu[1*], Xiaobo Yang[1*], Chaolin Huang[2*], Xiaojing Zou[1*], Ting Zhou[1*], Shangwen Pan[1*], Luyu Yang[3], Yongran Wu[1], Yaqi Ouyang[1], Yaxin Wang[1], Dan Xu[1], Xin Zhao[1], Huaqing Shu[1], Yongxiang Jiang[1], Wei Xiong[1], Lehao Ren[1], Hong Liu[1,2], Yin Yuan[1], Hong Qi[1], Shouzhi Fu[3], Dechang Chen[†], Dingyu Zhang[2†], Shiying Yuan[1†], You Shang[1,2†]

Additional file 1.

Figure S1. Kaplan–Meier plots showing the survival probability of those patients who received MV before and after 48 hours of HFNC initiation.

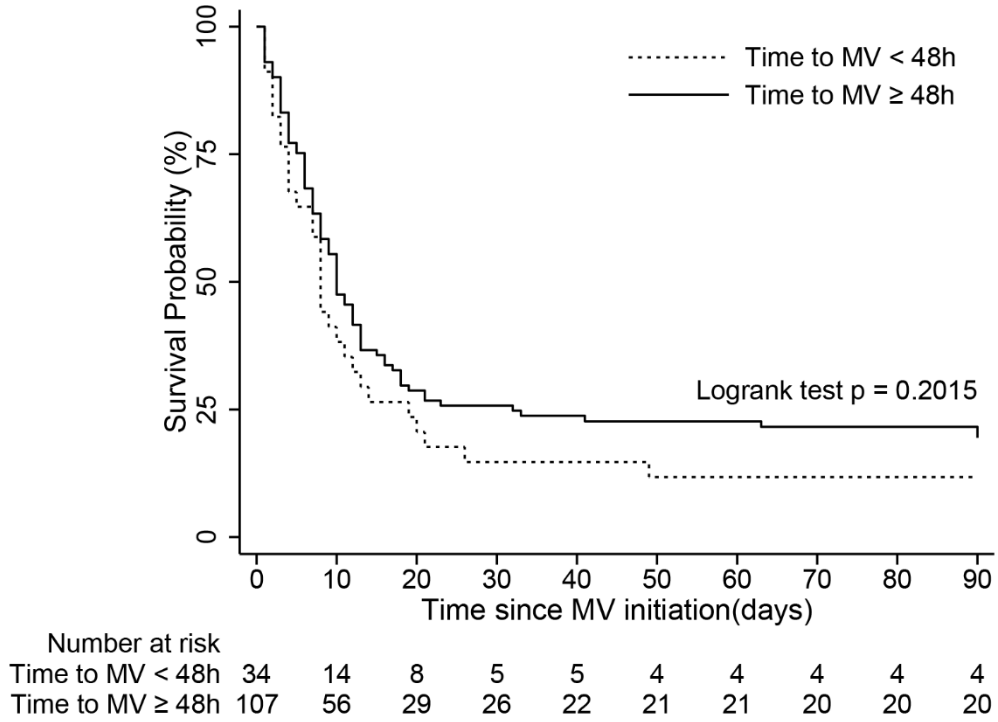

Abbreviations: COVID-19, coronavirus disease 2019; MV, mechanical ventilation.

Additional file 2.

Figure S2. Relative risk of death according to the time of MV in COVID-19 patients who failed on high-flow nasal cannula.

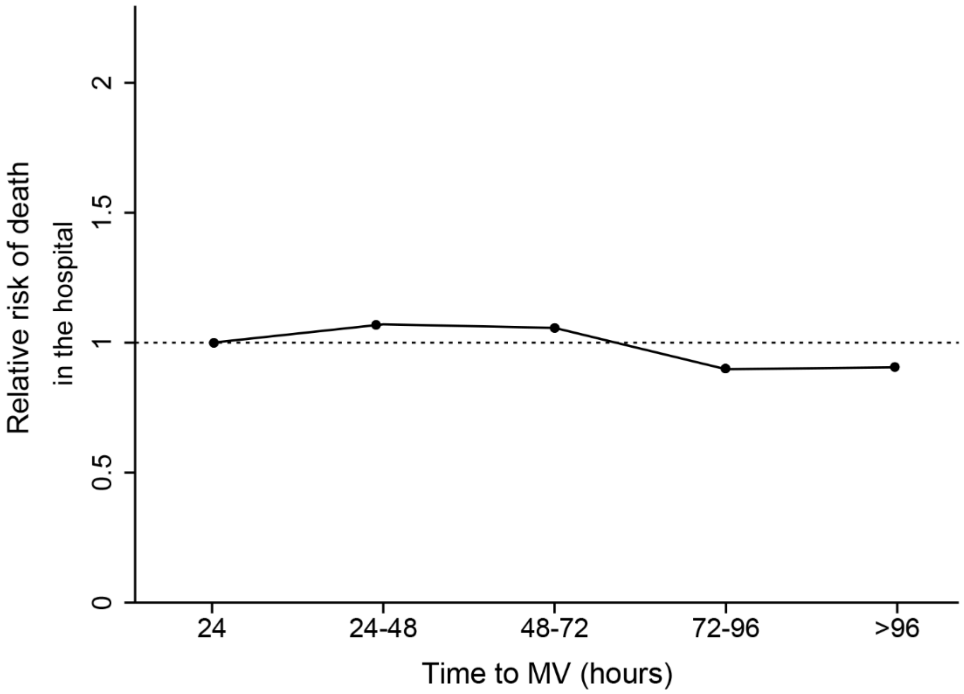

Abbreviations: COVID-19, coronavirus disease 2019; HFNC, High-flow nasal cannula; MV, mechanical ventilation.

Additional file 3.

Table S1. Baseline characteristics of the study cohort at HFNC initiation

**Characteristics**	**Train cohort (n=324)**	**Validation cohort (n=69)**	***p* Value**
Age, mean ± SD, years	63.2± 14.5	63.6 ±12.3	0.9939
Age >= 60 years	211 (65.1%)	45 (65.2%)	0.553
Male	219 (67.6%)	42 (60.9%)	0.175
**Preexisting comorbidities**	232 (71.6%)	44 (63.8%)	0.126
Hypertension	147 (45.4%)	32 (46.4%)	0.491
Diabetes	60 (18.5%)	11 (15.9%)	0.378
Chronic Cardiac disease	42 (13.0%)	10 (14.5%)	0.430
Chronic pulmonary disease	26 (8.0%)	7 (10.1%)	0.354
Chronic liver disease	27 (8.4%)	2 (2.9%)	0.089
Cerebrovascular disease	25 (7.7%)	8 (11.6%)	0.203
Malignancy	15 (4.6%)	1 (1.5%)	0.195
Time from illness onset to HFNC, days	11 [8 -15]	11 [7 -14]	0.6454

Time from admission to HFNC, days	2 [0 - 5]	2 [0 -4]	0.3523
SOFA at HFNC onset[¶]	4 [2 - 5]	4 [3 - 5]	0.2267
**Laboratory findings at HFNC initiation**			
Platelets	187 [140.5 –246.5]	165 [125 – 221]	0.1029
Platelet count < 125 × 10^9 /L[‡]	65 (20.1%)	17 (24.6%)	0.243
D-dimer, µg/mL	3.3 [1.0 – 14.1]	2.3 [1.1 – 15.3]	0.4837
IL-6[‖], pg/mL	9.7 [7.0 - 14.5]	8.7 [6.7 – 11.2]	0.07
**Complications at HFNC initiation**			
Shock	2 (0.62%)	1 (2.2%)	0.329
AKI	37 (11.4%)	10 (14.5%)	0.297
Acute cardiac injury	99 (30.6%)	22 (31.9%)	0.466
Liver dysfunction	189 (58.3%)	37 (54.4%)	0.322
Coagulopathy	30 (9.3%)	9 (13.0%)	0.226
**Corticosteroids Treatment**			
Corticosteroids	214 (66.1%)	78 (60.9%)	0.074
Corticosteroids treatment onset in HFNC	171 (52.8%)	32 (46.4%)	0.202
Corticosteroids treatment onset prior to	92 (28.4%)	16 (23.2%)	0.235

HFNC			
ICU admission	206 (63.6%)	41 (59.4%)	0.302
Length of ICU stay, days	12 [6 - 22]	13 [8- 19]	0.9761
Length of hospital stay, days	19 [11 - 35]	19 [13- 26]	0.9975
**Mortality since HFNC initiation**			
14-day	151 (46.6%)	32 (46.4%)	0.540
28-day	154 (47.5%)	33 (47.8%)	0.535
60-day	168 (51.9%)	34 (49.3%)	0.399
90-day	177 (54.6%)	35 (50.7%)	0.306

Abbreviations: AKI, acute kidney injury; COVID-19, coronavirus disease 2019; IQR, interquartile range; SD, standard deviation; HFNC, High-flow nasal cannula; IL-6, interleukin 6; SOFA, Sequential Organ Failure Assessment;

Data were expressed median [interquartile range] or as mean ± standard deviation.

[⁋]SOFA scores at HFNC imitation were available in 218 patients of train cohort and 49 patients of validation cohort.

[†]The upper limit of normal range of D-dimer was 1.5μg/mL.

[‡]The lower limit of normal range of platelet count was 125× 10^9/L.

[¶]The upper limit of normal range was 7 pg/ml.

Additional file 4.

Table S2. Physiologic Variables of the study cohort at HFNC initiation and within the first 4 hours of HFNC initiation

**Parameters**	**Train cohort (n=324)**	**Validation cohort (n=69)**	***p* Value** Success vs failure
Heart rate at HFNC initiation	88.8± 16.3	92.0 ±116.3	0.2969
Systolic arterial pressure at HFNC initiation	129.2± 17.4	127.0±17.7	0.4952
Arterial blood gas analysis at HFNC initiation[⁋]			
pH	7.47 ± 0.05	7.46 ± 0.05	0.2781
$PaCO_2$, mmHg	34.3±7.7	36.0±7. 9	0.1135
HCO_3^-, mmol/L	25.1 ± 5.4	26.0 ± 4.7	0.3337
PaO_2/FiO_2, mm Hg	143.1 [115.6 – 192.5]	140.0 [109.8- 177.8]	0.6101
<200 mm Hg	174 (79.8%)	40 (81.6%)	0.474
Respiratory parameters at HFNC initiation			
SpO_2	86 [80 -90]	89 [80 -92]	0.0579
SpO_2/FiO_2	108.7 [91.6- 143.3]	112.5 [90.5 – 133.3]	0.987
RR (beats per minute)	25 [22 - 30]	26 [21 - 30]	0.4975
ROX index	4.3 [3.4 - 5.5]	4.4 [3.5 – 5.7]	0.6705

After HFNC treatment			
SpO_2	94 [90 -96]	94 [90-96]	0.6803
SpO_2/FiO_2	117.5 [103.9 -142.2]	117.5 [95.0 -134.3]	0.0604
RR (beats per minute)	22 [21 - 24]	23 [21 - 25]	0.1511
Flow (L/min)	55.0 [50.0 - 60.0]	50.0 [40.0 - 60.0]	0.1557
ROX index	5.23 [4.59 – 6.35]	5.59 [4.2 -5.92]	0.1175
Length of HFNC, median [IQR], days	6.0 [3.0 - 11.0]	6 [3.0 – 11.0]	0.3524

Abbreviations: COVID-19, coronavirus disease 2019; FiO_2, fraction of inspired oxygen; IQR, interquartile range; PaO_2, partial pressure of oxygen; $PaCO_2$, partial pressure of carbon dioxide. ROX, Respiratory rate-oxygenation; SD, standard deviation;

Data were expressed median [interquartile range] or as mean ± standard deviation.

[a]Arterial blood gas analysis was conducted in 218 patients of train cohort and 49 patients of validation cohort.
